# Treatment preferences amongst physical therapists and chiropractors for the management of neck pain: results of an international survey

**DOI:** 10.1186/2045-709X-22-11

**Published:** 2014-03-24

**Authors:** Lisa C Carlesso, Joy C MacDermid, Anita R Gross, David M Walton, P Lina Santaguida

**Affiliations:** 1 Toronto Western Research Institute, University Health Network, 399 Bathurst Street - MP11-328, Toronto, Ontario M5T 2S8, Canada; 2 School of Rehabilitation Sciences McMaster University, Hamilton, Ontario, Canada; 3 Clinical Research Lab, Hand and Upper Limb Centre, St. Joseph’s Health Centre, London, Ontario, Canada; 4 School of Physical Therapy, Western University, London, Ontario, Canada; 5 Department of Clinical Epidemiology and Biostatistics, Hamilton, Ontario, Canada

**Keywords:** Survey, Neck pain, Treatment, Practice patterns, Complementary and alternative medicine

## Abstract

**Background:**

Clinical practice guidelines on the management of neck pain make recommendations to help practitioners optimize patient care. By examining the practice patterns of practitioners, adherence to CPGs or lack thereof, is demonstrated. Understanding utilization of various treatments by practitioners and comparing these patterns to that of recommended guidelines is important to identify gaps for knowledge translation and improve treatment regimens.

**Aim:**

To describe the utilization of interventions in patients with neck pain by clinicians.

**Methods:**

A cross-sectional international survey was conducted from February 2012 to March 2013 to determine physical medicine, complementary and alternative medicine utilization amongst 360 clinicians treating patients with neck pain.

**Results:**

The survey was international (19 countries) with Canada having the largest response (38%). Results were analyzed by usage amongst physical therapists (38%) and chiropractors (31%) as they were the predominant respondents. Within these professions, respondents were male (41-66%) working in private practice (69-95%). Exercise and manual therapies were consistently (98-99%) used by both professions but tests of subgroup differences determined that physical therapists used exercise, orthoses and ‘other’ interventions more, while chiropractors used phototherapeutics more. However, phototherapeutics (65%), Orthoses/supportive devices (57%), mechanical traction (55%) and sonic therapies (54%) were *not* used by the majority of respondents. Thermal applications (73%) and acupuncture (46%) were the modalities used most commonly. Analysis of differences across the subtypes of neck pain indicated that respondents utilize treatments more often for chronic neck pain and whiplash conditions, followed by radiculopathy, acute neck pain and whiplash conditions, and facet joint dysfunction by diagnostic block. The higher rates of usage of some interventions were consistent with supporting evidence (e.g. manual therapy). However, there was moderate usage of a number of interventions that have limited support or conflicting evidence (e.g. ergonomics).

**Conclusions:**

This survey indicates that exercise and manual therapy are core treatments provided by chiropractors and physical therapists. Future research should address gaps in evidence associated with variable practice patterns and knowledge translation to reduce usage of some interventions that have been shown to be ineffective.

## Background

Clinical practice guidelines (CPG) are developed to provide statements and recommendations with the intention of helping practitioners optimize patient care [1]. By examining the practice patterns of practitioners, adherence to CPGs or lack thereof, is demonstrated. Recommendations for practice can then be formed. Understanding existing practice patterns provides insight into how current evidence impacts on practice and can identify where greater efforts in knowledge translation are needed. Clinical practice will vary dependent on a number of factors such as location, resources available, patient population, and professional background. Several CPGs from varying professionals who treat patients with neck pain exist [2-5]. To our knowledge no examination of practice patterns across health care professionals who treat patients with neck pain has been published.

Neck pain is a common problem with an episodic course that affects a large proportion of the population. Estimates for the prevalence of neck pain vary from 0.4% to 86.8% (mean 23.1%) in the general population, whereas the range for the one year incidence is reported to be smaller (10.4% to 21.3%) [6]. Risk factors for new onset neck pain include being female, between the ages of 35 to 49 years and having a previous episode of neck pain [6-8]. Estimated expenditures on spine related care in the United States have almost doubled in the last decade [7,9]. The number of emergency room visits related to motor vehicle accidents (MVA) has been steadily increasing in the last three decades [10]. Direct healthcare costs may only be a small piece of this burden, while the indirect costs of work absenteeism and disability are much greater [6,11].

Over the past decade the evidence base guiding the choice of effective treatments for reducing symptoms and increasing function has evolved. The association between severity and disability of neck pain has been established by numerous studies [12-15]. There are numerous health care practitioners that treat patients with neck pain with a variety of interventions such as physical medicine, manual therapies, exercise, electrotherapeutic agents, and ergonomics [16]. Manual therapies in combination with exercise may provide optimal treatment effects [17]. Outcomes such as better pain reduction, better patient satisfaction, improved function, increased range of motion and increased strength in people with neck pain have been reported in patients who received manual therapy alone or in combination with other modalities [16,18-22]. While evidence exists for other interventions, the number of studies is few with conflicting results leading to less confidence in their effectiveness [16,23-25].

Our recent reviews of reviews [26,27] provide some guidance for practitioners. There is moderate evidence for specific modalities such as laser and acupuncture for pain reduction while there is evidence of no benefit for the use of collars, ergonomic or physical environment changes in the workplace. Ideally, providers make treatment choices based on their experience, the available evidence, and the clinical presentation of their patient.

In reality however, provider choices of care may be influenced by factors such as scope of practice, patient preferences, types of providers the patient chooses to see or insurance coverage, thereby influencing the types of treatments received and by their interaction with providers. Even within the same profession, providers’ choices of care may vary depending upon the degree to which they are aware of current evidence on treatment effectiveness and the degree to which they choose to incorporate the evidence into their practice. For example, care provided by a physical therapist will differ, in some ways from care provided by a physician or from another physical therapist. Patient centered care and the shared decision making model have incorporated patient preferences into treatment planning. This denotes the importance of flexibility in the way that health professionals structure the decision-making process so that individual patient differences can be respected [28]. The incorporation of patient preferences has the potential to shift ‘ideal’ choices to more practical ones based on preferences of each individual. For example, a patient treated previously for a different problem and presenting with a new problem may have had a successful outcome using an evidence based intervention. However applying the same intervention to a different area may not be supported. Given the favourable outcome, the patient may request that the same intervention be used. The practitioner is therefore faced with making treatment decisions that incorporate patient expectation and it has been suggested that doing so may improve adherence and satisfaction with care [29]. The end result is more varied care and a modification of the ‘ideal’ treatment allowing incorporation of patient preferences and expectations but straying slightly from recommended guidelines.

There is little evidence in the literature about guideline adherence in patients with neck pain. A study by Oostendorp et al. (2013) used quality indicators of treatment based on guideline recommendations for patients with non specific neck pain [30]. The study by Oostendorp et al. used a sample of 38 physiotherapists and 96 patients in the Netherlands. Adherence to the identified treatment quality indicators ranged from weak (34%) to adequate (59%). These findings provide some initial insight into the potential variability in care that may exist for this patient population and practitioner group but does not provide details about the types of interventions or the frequency with which they were used.

The International Collaboration on Neck Pain (ICON) project is a collaborative project amongst internationally recognized experts in the field of neck pain. The goal of ICON is to establish clear, actionable messages in the areas of diagnosis, prognosis, interventions and outcomes measurement. To establish such data, ICON has implemented an international multidisciplinary survey of clinical practice patterns that will help shape evidence based recommendations.

The purposes of this study were 1. To describe the utilization of interventions in patients with neck pain by clinicians and 2. Where appropriate, to examine whether utilization varies by profession or subgroup of disorder.

## Methods

A cross sectional survey to determine practice patterns of clinicians who provide care to patients with neck pain was conducted from February 2012 to March 2013. The survey was approved by the McMaster University Research Ethics Board (REB#11-025).

### Survey development and validation

The survey was designed to acquire information in four principle content areas. These four areas included examination/diagnostic procedures, prognostic indicators, interventions (including adverse events), and outcome measures. Two additional content areas determined the demographic and caseload information of respondents.

The survey was developed using Streiner and Norman (2008) [31] methods in three distinct and iterative steps – item generation using literature review, consultation with clinicians which included clinical observation, and ICON content experts - before fielding the questionnaire. 1) The initial core set of items was generated from systematic reviews on conservative treatments for neck pain. 2) This set was then sent to expert clinicians identified within and external to ICON for review of usability, technical functionality of the electronic questionnaire and identification of issues/gaps. 3) The next set was tested using the ICON expert panel of interdisciplinary professionals; this included physicians, psychologists, physiotherapists, massage therapists, chiropractors, and other rehabilitative professions. Additional items created by these experts aimed to address areas missing from the initial item generation to ensure they were designed to be appropriate for administration across different health care professionals commonly treating people with neck pain.

This content area being sufficiently large was divided into the following two separate surveys: 1. Physical medicine or CAM interventions; and 2. Pharmacological and psychological interventions. Items in the physical medicine and CAM survey covered exercise, manual therapies, modalities, mechanical traction, orthoses/supportive devices, ergonomic and work related interventions. An ‘other’ response option was included where appropriate within each category and allowed respondents to add any interventions that may have been missed. Broadly, items asked about utilization of each treatment category (yes, no, outside scope of practice). The questions progressed in the following sequence. If a respondent indicated ‘yes’ to the initial utilization question, then inquiry of frequency of use (commonly, occasionally, never) followed. If respondents indicated ‘common’ or ‘occasional’ use of an intervention, then use of that intervention (commonly, occasionally, never, not applicable) among the following common subgroups of neck pain disorders was also inquired about:

1. Acute nonspecific neck pain,

2. Chronic nonspecific neck pain,

3. Facet joint dysfunction (as diagnosed by diagnostic block),

4. Acute whiplash associated disorder (WAD),

5. Chronic WAD and

6. Radiculopathy/WADIII.

For the modalities category, respondents were also asked about indications for use. Routing questions were sequentially designed to reduce respondent burden allowing avoidance of categories not relevant to respondents.

Face and content validity of the survey were addressed in an iterative process involving multiple revisions and content experts. In the first round, there was a focus on accuracy of item content and clarity in the wording of each item and response. In the second round, there was a focus on the organizational structure of the survey so as to have logical grouping, sequencing of items and routing questions. In the third round, responses were piloted within the electronic survey format to evaluate presentation and routing. Finally, the ICON working group (n = 38) that had representation from all the disciplines included in our target audience was used for field-testing. These experts reviewed the survey for accuracy, clarity, completeness and burden. After each round of revisions, editing occurred for clarity. This resulted in minor changes to items and a few additions. The finalized version was mounted using LimeSurvey^a^, a software program for web-based survey administration.

### Sampling frame

Our sampling frame was all health care professional groups identified as having a major role in the management of neck pain, relying on both our reviews and clinical experience to identify these groups. This included physicians (general practitioners, physiatrists, psychiatrists) physiotherapists, chiropractors, massage therapists, occupational therapists, psychologists and complementary medicine specialists. We wanted to include an international perspective. Given the number of national associations and a general lack of willingness for professional associations to burden their members with survey requests we relied on Snowball sampling strategies.

This method allowed clinician experts identified by ICON within each of the professions. Invitations were sent to the contact person identified for each association and requested they assist with sending out links to our survey to their professional links and colleagues. If the association responded and agreed to send out the link on our behalf, it was done so at their discretion. In total 37 groups were contacted, 19 of which did not respond. Survey invitations were distributed via e-mail blast to members, and electronic postings (e.g., e-newsletter, website, Facebook or Twitter pages) by national and international professional groups for chiropractors (Danish Chiropractors’ Association; European Academy of Chiropractic; Netherlands Chiropractic Association; New Zealand Chiropractors’ Association; Ontario Chiropractic Association); manual therapists (Canadian Academy of Manipulative Physical Therapy; Dutch Association for Manual Therapy; Finnish Association for Orthopedic Manual Therapy; German Manual Therapy Journal; International Federation of Orthopaedic Manipulative Physical Therapists); massage therapists (Massage Therapists’ Association of British Columbia); physicians (North American Spine Society; University of British Columbia Department of Family Medicine); physiotherapists (American Physical Therapy Association – Orthopedic Section; Canadian Physiotherapy Association – Pain Sciences Division; Hong Kong Physiotherapy Association; Musculoskeletal Physiotherapy Australia; Physiopedia); and other health care professionals (Osteopathic Society of New Zealand). The method of recruitment meant we were unable to determine how many people received our requests for participation. We were unaware if the associations sent the survey link to all members or only those who agreed to receive it.

### Survey administration

The survey was administered through the International Collaboration on Neck (ICON) group and was estimated to take 15-20 minutes to answer. An e-mail including information about the survey, and a registration link were provided. No incentives were offered. Public registration was required to participate in the survey and individuals who volunteered to receive the survey link were considered “registrants”. Once respondents registered, an email containing the link to participate in this survey was sent out immediately. Respondents remained anonymous by storing the identification tokens (name and e-mail address) that provided access to the survey on a secure separate database. Registrants were notified that clicking the survey link indicated that they were electronically consenting to participate. Weekly reminders were sent to registrants until they completed the survey, opted out, or received a maximum of four reminders. Response rates amongst registrants were calculated based on the number of registrants who completed at least part of the survey.

### Analysis

Data quality was assessed by randomly sampling 10% of the dataset to check for errors. Discrepant entries (< 1%) were resolved through this process. Descriptive statistics were used to summarize participants and their responses to each question. Chi-squared analyses were used to test for differences in the frequency of use of various treatment interventions based on profession. Rank order was used to assess frequency of use of interventions amongst subtypes of neck pain.

## Results

There were 360 respondents (332 full and 28 partial) spanning 17 countries. Respondents were mainly physical therapists (38%) or chiropractors (31%) and largely male (41-66%). Due to lack of adequate representation from professions other than physical therapists and chiropractors, we focused the analyses on these two groups. Based on the relevant physiotherapy and chiropractic professional associations’ membership information that we were able to ascertain, as many as 17773 individuals were invited to respond to our survey. This is likely a high estimate as more detailed information from a few organizations indicates that variation existed due to inactive emails, unsent links (one international body represents 22 countries) and membership number fluctuations throughout the year. Using this very conservative total, results in a response rate of approximately two percent. Table 1 provides the characteristics for the whole sample and physical therapists and chiropractors only. Within the two professions, there was an average of 15 years of clinical experience, and 34% had obtained a Master’s degree. The majority (80%) worked in private practice, with reimbursement through private insurance (79%) in a fee for service private payment model (64%). Over half of the respondents indicated that patients with neck pain made up at least one quarter of their caseload. The largest subset of respondents was from Canada (38%). The gender distribution of this subset of respondents is representative of both professions (males 41% physical therapists; 66% chiropractors) [32,33].

**Table 1:** Demographics

**Demographics**	**Respondents**	**Respondents (n = 251)**	
	**(n = 360)**	**Chiropractor**	**Physical therapist**
		**(n = 113)**	**(n = 138)**
Years in practice since graduation (mean (sd))	16 (12)	16 (12)	16 (12)
Gender	44% female, 48% male	35% female, 66% male	59% female, 41% male
**Profession**			
Physical therapist (Manual therapist)	38% (13%)	-	55%
Chiropractor	31%	45%	-
Massage therapist	9%	-	-
Physician	5%	-	-
Other	14%	-	-
**Country**			
Canada	38%	56%	23%
United Kingdom	10%	3%	19%
United States of America	10%	1%	12%
Denmark	9%	26%	-
New Zealand	9%	1%	11%
Netherlands	6%	3%	9%
Other (Australia, Belgium, Brazil, Finland, Germany, Hong Kong, Italy, Norway, Portugal, South Africa, Sweden,)	18%	13%	25%
**Practice setting**			
Private clinic	72%	95%	69%
General hospital	7%	1%	14%
Teaching hospital	7%	3%	10%
Outpatient rehab facility	7%	6%	9%
Private consultant	6%	3%	7%
Other	9%	4%	12%
**% of caseload with neck pain**			
<5	3%	0%	3%
6-20	21%	14%	34%
21-50	47%	60%	49%
>50	24%	26%	15%
**Health care reimbursement system**			
Private insurance	72%	83%	75%
Public health insurance	45%	40%	51%
Workers compensation	36%	40%	36%
**Salary reimbursement scheme**			
Salary - Fixed	27%	17%	46%
Fee for service – Public	22%	27%	12%
Fee for service – Private	61%	75%	55%
**Education - Highest level obtained**			
Diploma	9%	4%	5%
Bachelor’s degree	19%	6%	33%
Masters degree	29%	*	41%
Doctor of medicine	4%	3%	1%
Doctorate/PhD	16%	27%	15%
Other	14%	35%	4%
		(Doctor of chiropractic)	

*This value for chiropractors is not reported as it overlaps with the ‘Other’ category.

Table 2 demonstrates the frequency of use (‘Yes’) of the various physical medicine and CAM treatment interventions along with their subtypes. Exercise prescription was used at least by 98% of respondents, as were manual therapies. Ergonomic advice (83%), work related interventions (73%) and thermal agents (73%) rounded out the top 5 most frequently endorsed treatment approaches. Respondents indicated that they largely did not provide (‘No’) ‘other’ interventions (70%), phototherapeutics (65%), orthoses/supportive devices (57%), mechanical traction (55%), sonic therapies (54%) or electrotherapeutics (47%).

**Table 2:** Provision of treatment interventions

**Interventions**	**Yes %**	**Yes commonly %**	**Yes occasionally %**	**Yes never %**	**No %**	**Outside scope of practice**
**Exercise**	98				2	0
Stretch neck/upper thorax		79	17	2		
Stretch other body part		47	43	8		
Strengthen neck/upper thorax		77	19	3		
Strengthen other body parts		51	41	7		
Local muscle endurance		63	26	9		
Postural control		84	13	2		
Exercises related to motor control		40	41	18		
Static/dynamic stabilization		55	32	12		
Cardiovascular retraining		25	51	23		
Other:		17	53	29		
**Mechanical Traction**	28				55	12
**Modalities**						
*Electrotherapeutics*	43				47	9
TENS		12	27	4		
EMG biofeedback		1	9	26		
Short wave diathermy		0	5	31		
Muscle stimulation		5	13	19		
*Thermal agents*	73				22	5
Heat or cold application		55	13	0		
*Phototherapeutics*	10				65	23
Laser therapy		5	4	1		
*Sonic therapies*	29				54	17
Ultrasound		11	13	0		
Shock wave		0	2	21		
*Acupuncture*	46				29	25
Traditional acupuncture		17	17	7		
Dry needling		18	11	12		
**Manual therapies**	99				1	0
Mobilization		90	8	1		
Manipulation		56	32	11		
Manual traction		55	37	7		
Massage/soft tissue work		79	19	1		
**Orthoses/supportive devices**	30				57	11
Collars		0	24	5		
Pillows		8	20	3		
Taping		4	16	10		
Adaptive equipment		0	5	25		
Other		0	1	28		
**Ergonomic interventions**	83				15	8
**Work related interventions**	73				22	9
Work hardening		8	35	29		
Work site modification		33	36	3		
Communication with employer		16	43	13		
Work site restrictions		19	47	7		
Other		7	23	42		
**Other**	13				70	6

Different types of exercise were more *commonly* used such as those for postural control (84%), stretching of the neck/upper thorax (79%) and strengthening of the neck/upper thorax (77%). The exceptions were motor control (41%) and cardiovascular training (51%), which were used more *occasionally*. The most *commonly* used modalities included hot and cold applications (55%) and acupuncture (traditional 17%, dry needling 18%). A substantial number of respondents rarely or never used short wave diathermy (31%), biofeedback (26%), shock wave sonic therapy (21%) and muscle stimulation (19%). The majority of respondents *commonly* used manual therapies and this was most frequently mobilization (90%) compared to manipulation (56%). Work related interventions such as work hardening, site modification, communication with the employer and site restrictions were used *occasionally* (35-47%).

Table 3 highlights the use of modalities and their indications for use. The only two modalities that the majority of respondents indicated that they used were Thermal applications and TENS. Both were indicated for pain relief, 90% and 71% respectively. For the remaining modalities, for all but ultrasound and muscle stimulation (45% each), the majority of respondents indicated that they ‘Do not use/outside scope of practice’.

**Table 3:** Indications for use of modalities

	**Pain relief**	**Retrain or strengthen muscle**	**Enhance tissue healing**	**Alter tissue prior to MT**	**Do not use/outside scope of practice**
TENS	71%	3%	5%	7%	26%
EMG biofeedback	2%	28%	1%	1%	66%
Short wave diathermy	5%	0%	4%	5%	83%
Muscle stimulation	16%	42%	11%	15%	45%
Heat or cold application	90%	1%	34%	42%	1%
Laser therapy	12%	1%	28%	3%	65%
Ultrasound	25%	1%	40%	18%	45%
Shock wave	1%	1%	8%	1%	86%
Traditional acupuncture	37%	5%	18%	12%	55%
Dry needling	34%	4%	15%	21%	55%

### Subgroup analysis

#### Provider

**Figure 1:**
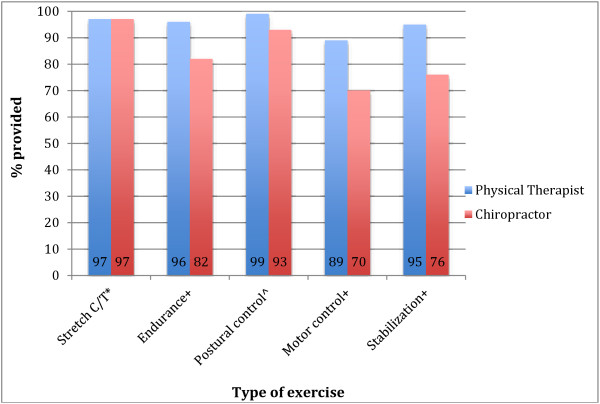
Significant differences between professions in exercise prescription.

**Figure 2:**
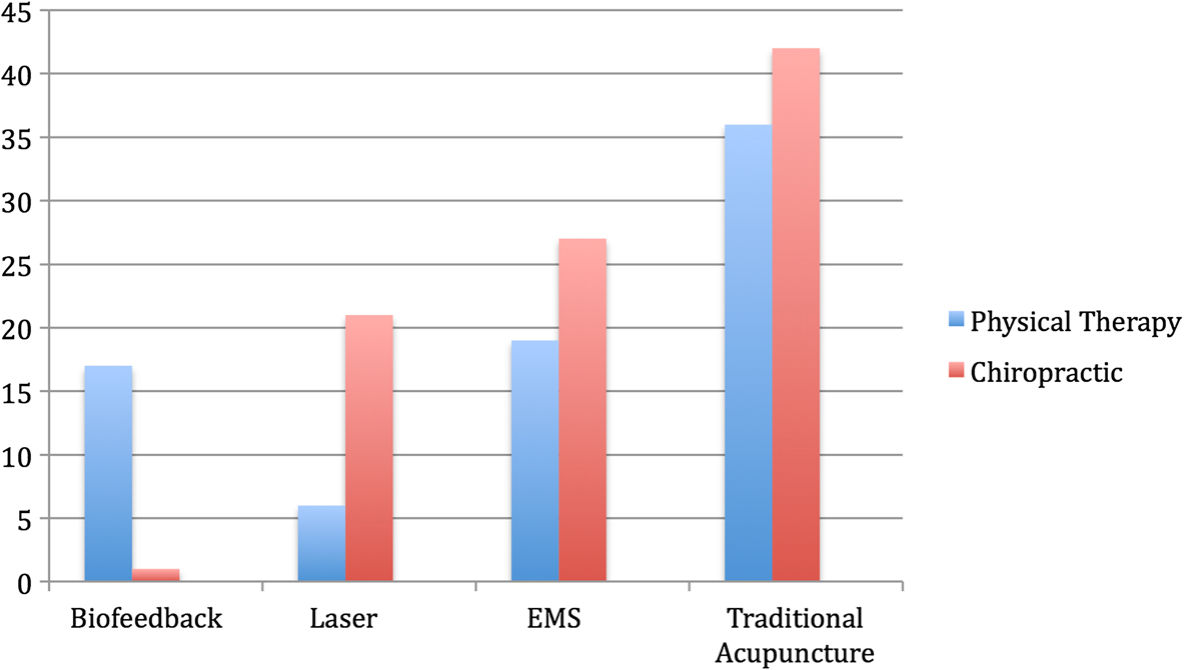
Significant differences between professions in modality use.

Looking across physical therapists and chiropractors, differences in their use of the various interventions were found. Table 4 shows that the two differ in their prescription of exercise, phototherapeutics, orthoses/supportive devices and the category of ‘other’ interventions. Both collars and taping are used more often by physical therapists than chiropractors (p = 0.01 and p = 0.03 respectively). Examples of ‘other’ interventions used more frequently by physical therapists include pain education, referral to other healthcare professionals, use of McKenzie methods [34], self-management strategies, breathing/relaxation strategies and prolotherapy. Figure 1 depicts the differences in exercise prescription between the two. Significant differences were found in the prescription of some types of exercise (greater by physical therapists p ≤ 0.01) except stretching of other body parts, strengthening of neck/upper back and other body parts and cardiovascular training where there was no difference. Figure 2 shows the differences in use of modalities. Chiropractors use of laser (p = 0.00), and electrical muscular stimulation (p = 0.01) was significantly greater than physical therapists. Although there is no significant difference between the two in the overall use of manual therapies, there is a significant difference in the use of thrust manipulation (p = 0.00) with chiropractors performing it more often.

**Table 4:** Differences in provision of treatment interventions across professions

**Interventions**	**Physical therapy versus chiropractic significance and direction**
Exercise	p = 0.01, PT–138 CH–107
Electrotherapeutics	No difference
Thermal agents	No difference
Phototherapeutics	p = 0.00, CH–23 PT–10
Sonic therapeutics	No difference
Acupuncture	No difference
Manual therapies	No difference
Mechanical traction	No difference
Orthoses/support devices	p = 0.05, PT–53 CH–26
Ergonomic interventions	No difference
Work related interventions	No difference
Other	p = 0.00, PT–29 CH–4

*PT* = Physical therapist, *CH* = Chiropractor.

### Disorder subgroup

Table 5 outlines the use of treatment interventions according to disorder subgroup. Respondents reported using exercises, manual therapy, and ergonomic interventions most frequently in patients with chronic nonspecific neck pain compared to the five other conditions. Differences between chronic nonspecific neck pain and chronic WAD were minimal. The use of orthoses/supportive devices was variable across the 6 subgroup disorders. Respondents indicated that collars were used most frequently for patients with acute WAD (24%), pillows for patients with chronic nonspecific neck (27%), while the use of taping (16%) was split across three subgroups and adaptive equipment (5%) in chronic non specific neck pain. Utilization of three of the four work related interventions was equal in patients with chronic non specific neck pain or chronic WAD (43 to 68%). Respondents indicated that they used mechanical traction most frequently for patients with radiculopathy or WAD III (27%). Rank ordering of interventions within the subgroups from most utilized to least utilized resulted in the following: 1. chronic nonspecific neck pain; 2. chronic WAD; 3. radiculopathy; 4. acute nonspecific neck pain; 5. acute WAD; and 6. facet joint dysfunction by diagnostic block. This would indicate that chronic conditions are being treated with the interventions we inquired about more frequently and that respondents are also utilizing a wider range of interventions.

**Table 5:** Provision of treatment interventions by subgroups

	**Acute non specific NP + %**	**Chronic non specific NP%**	**Facet joint dysfunction by diagnostic block %**	**Acute WAD%**	**Chronic WAD%**	**Radiculopathy (WAD III)%**
**Exercise**						
Stretch neck/upper thorax	78	**97**	50	63	92	65
Stretch other body part	69	**86**	42*	63	83	65
Strengthen neck/upper thorax	69	**94**	51	62	92	75
Strengthen other body parts	61	**87**	47	57	86	65
Local muscle endurance	63	**87**	51	59	86	65
Postural control	84	**95**	57	81	92	85
Exercises related to motor control	56	**72**	40*	56	71	59
Static/dynamic stabilization	68	**84**	48	67	83	73
Cardiovascular retraining	29*	**57**	26*	31*	56	38
**Manual therapies**						
Mobilization (joint or neuromuscular)	95	**97**	61	86	95	89
Manipulation (thrust)	73	**82**	51	51	77	49
Manual traction	82	**83**	51	66	77	79
Massage/soft tissue work	93	**95**	58	85	92	84
**Mechanical traction**	17	25	15	12	24	**27**
**Orthoses/supportive devices**						
Collars	21	6*	6*	**24**	8*	20
Pillows	22	**27**	18	23	26	23
Taping	**16**	**16**	10	14	**16**	14
Adaptive equipment	3	**5**	2	3	4	4
Other	2	1	1	2	1	1
**Ergonomic interventions**	73	**83**	49	70	81	75
**Work related interventions**						
Work hardening	19*	**43**	21*	21*	**43**	27
Work site modification	56	**68**	37	57	**68**	63
Communication with employer	45	**55**	7*	50	**55**	52
Work site restrictions	59	55	31*	**61**	58	61
Other	21	**22**	13*	20	21	21
**Other types of interventions**	12	**15**	8*	11	14	12

+NP = Neck Pain.

* = percentage is ≤ to respondents indicating never use/not applicable.

Bolded = highest percentage amongst subgroups.

## Discussion

### Summary of main findings

Our results from this survey demonstrate that the interventions most commonly used by physical therapists and chiropractors for the treatment of people with neck pain (exercise and manual therapy) are those that also have strong evidence for their effectiveness, [17,35-38] particularly when combined. It is these two interventions that are also recommended by clinical practice guidelines [2,4]. Other treatments do not reflect the best evidence for effective treatment. The results also indicate variable use of interventions with low or very low evidence. Some are being used to a larger degree (ergonomic and work related interventions) [39,40] while others are not being used (mechanical traction, orthoses/supportive devices and modalities) [23,24]. Other treatments may be used more as they address immediate or short term symptom relief but they lack sufficient focus on long-term functioning [17,27].

### Exercise

A Cochrane review of exercise interventions for patients with neck pain highlighted that a combination of cervical and scapulothoracic stretching and strengthening improves pain and function and leads to greater patient satisfaction in people with chronic neck pain. Endurance exercises of the cervical and scapulothoracic regions are effective at reducing pain, improving function and global perceived effect for subacute/chronic cervicogenic headache, while neck strengthening is effective at reducing pain in acute cervical radiculopathy. Neither upper extremity stretching, strengthening or a general exercise program is recommended for chronic neck pain [35]. In this study, respondents’ reported the greatest utilization of exercise in chronic nonspecific neck pain and WAD conditions demonstrating that current practice patterns are in sync with evidence based recommendations. We did not have a specific category to capture cervicogenic headache so we cannot be certain of how this subgroup was considered. Our response options regarding exercise covered many general concepts ranging from stretching, strengthening, endurance, postural control, motor control and stabilization, but did not allow for details within these categories to be explored. Overall exercise prescription was reported most often in the two chronic neck pain conditions and radiculopathy, which is consistent with the findings of the systematic review.

### Manual therapies

Manual therapies are a core skill for the majority of respondents so it is not surprising that they are used as frequently as exercise. Mobilization and massage therapy/soft tissue work were the two most commonly reported of the four techniques. Possible explanations for this lie in our sample representing physical therapists and chiropractors as well as in the evidence base. A Cochrane review has reported the benefit of using mobilization techniques and that no differential benefit for spinal manipulation as opposed to mobilization has been shown for the outcomes of pain, function and patient satisfaction [36]. Using the techniques alone compared to combining them with exercise is mainly of short term benefit on pain only [37]. Regarding the effectiveness of massage therapy/soft tissue techniques, findings of systematic reviews are concordant [41-43]. Evidence indicates some pain reduction and improvement in function for subacute and chronic neck pain immediately post massage therapy/soft tissue treatment but there is inconclusive evidence of any long-term benefit. Despite the lack of clear direction in the literature, the proportion of utilization of massage therapy/soft tissue techniques was similar between the physical therapists and chiropractors in this survey. Massage/soft tissue techniques may be selected for a variety of reasons including complementary or preparatory effects prior to vertebral mobilization/manipulations [43] and the fact that patients report it being very helpful for neck pain [44,45]. It is possible that there are differences in the type of soft tissue techniques performed by the two different professions, but the nature of survey work does not allow for these distinctions.

### Ergonomics, work related interventions and Orthotics/ Supportive devices

The association between poor workstation design and awkward work postures with neck pain has been documented [46]. Recent systematic reviews reported limited evidence for decreased neck pain when physical ergonomic interventions were used compared to none at all [39,47]. The following interventions are reported to have no evidence of benefit: ergonomic education (for intervention), workplace physical environment changes (for intervention and primary prevention), and individual worker upper extremity stretching and endurance training program (for intervention, primary and secondary prevention) [26]. In the absence of strong evidence for ergonomic or work related interventions, and considering the difficulties and potential high costs of these interventions it may be that practitioners are less likely to select this treatment approach. Our survey does not allow us to determine why people select different interventions and thus whether it is perceived effectiveness, skills or resources that drive the lower utilization of ergonomic and workplace interventions. The lower rate of utilization of these interventions by respondents, particularly for complex patients like those with chronic WAD may reflect the challenge that practitioners face in addressing interventions for complex patients with limited evidence [48,49].

Overall, respondents provided little endorsement for the use of mechanical traction, and orthoses/supportive devices. However, responses suggest that there may be subgroups where clinicians feel these interventions could be beneficial. For example, mechanical traction was used most frequently in the radiculopathy/WAD III population compared to the other subgroups, despite inconclusive evidence for its use either intermittently or continuously [24]. This may suggest that practitioners expect that a force to unload pressure on the nerve is indicated in nerve root compression. Some evidence does exist for intermittent traction for generalized mechanical neck pain which may be influencing practitioner usage [50] and we have recently found evidence of moderate benefit for intermittent traction for chronic neck pain [27]. We did not ask about intermittent versus continuous application and therefore cannot be certain how this was interpreted.

Cervical pillows were used most commonly in chronic neck pain and WAD conditions while collar use was reported most often in acute non specific neck pain despite evidence of no benefit [51,52] or that collar use is detrimental to recovery [53]. In our concurrent review we found no evidence to support the effectiveness of soft collars in acute neck pain or WAD [26].

### Modalities

We did not inquire about modality usage by disorder subgroup but by indications for use. When implemented, the rationale selected by respondents for specific modalities was consistent with their accepted indications. TENS, Heat/cold and acupuncture were used for pain relief and laser therapy for tissue healing. The existing literature base shows moderate evidence supporting the use of these modalities for neck pain [16,23,54] with the exception of heat/cold where little evidence is present. A survey of patients in one state in the USA seeking care for neck pain reported that heat and cold therapy were received by 57% and 48% respectively compared to prescribed exercise (53%), spinal manipulation (37%) or TENS (22%) [55]. A survey of chiropractors in two states in the USA also reported use of thermal and electrical modalities more than exercise [56]. There may be several reasons for these discrepancies between the practice patterns reported in these two American studies versus the current findings. This could include factors such as: patient preferences, time management in busy clinics, clinicians balancing observations of treatment efficacy with the published evidence or perhaps being unaware of the current recommendations for care.

Our concurrent review of reviews indicates that acupuncture (short term pain relief) and low-level laser (short and intermediate term pain relief and function improvement) both have moderate evidence of benefit for chronic neck pain. Evidence of no benefit was found for pulsed ultrasound, or continuous traction [27]. Our data shows that respondents are practicing in line with the evidence in their minimal use of ultrasound and traction and greater use of acupuncture, but deviates from the evidence in their minimal use of low level laser.

### Disorder subgroups

Our data indicated some difference in treatment selection across different types of neck pain. Chronic neck pain conditions presented in our survey as non specific and WAD were more likely to be treated with physical medicine and CAM treatment interventions in comparison to radiculopathy/WAD III, (presented in our survey as acute neck conditions and facet joint dysfunction). Although it is tenuous to make assumptions about why respondents selected different interventions from this type of survey, it does appear that the pattern of utilization is consistent with variations in the complexity of the condition. Chronic neck pain conditions, particularly those arising following WAD [13,57] are often more complex, and associated with greater degrees of disability and impairment [57]. Our respondents indicated use of more varied interventions for chronic neck conditions compared to acute conditions, possibly in line with the complexity of the condition. Cervical nerve root pathology leading to upper extremity symptoms is often caused by space occupying lesions such as disc herniation, spondylosis, or osteophytosis that can be resistant to conservative treatment [58]. There is conflicting evidence around the efficacy of manual therapy, exercise, and other modalities for radiculopathy [3,58]. Acute neck pain and WAD if uncomplicated by high levels of pain severity, functional limitation or psychological distress will likely resolve within a reasonable timeframe. Our survey results seem to reflect this as these acute conditions had the lowest frequency of intervention utilization.

There is little published evidence on the effectiveness of conservative treatments for facet joint dysfunction confirmed by diagnostic block. The relative lack of evidence for treatment specific to this syndrome may be why respondents ranked it last of our listed disorders regarding overall frequency of utilization of interventions. Practitioners may suspect facet joint dysfunction after screening but it is likely that few are confirmed by diagnostic block thus making certainty of the diagnosis difficult.

### Differences and similarities between professions

Our findings indicate that some differences exist in the utilization of interventions between physical therapists and chiropractors. Although the scope of practice of these two professions is similar, these findings are not unexpected, as differences based on education and clinical paradigms could be expected that may influence their approach to treatment. Practitioner’s use of interventions can be shaped by factors other than their entry-level education such as courses taken post professionally, clinical environments, characteristics of the population treated, or use of evidence base medicine. The nature of the survey does not allow us to determine why differences existed between the professional groups. The higher utilization of exercise by physical therapists could reflect the fact that physical therapists have a strong focus on use of therapeutics exercise in their entry-level training or that there is a substantial body of therapeutic exercise evidence developed by physical therapists. We could anticipate that clinicians attend to research that is published within their own professional journals to a greater extent than literature from other disciplines [59-61]. Similarly, innovations in pain education have arisen in physical therapy literature and were cited in the ‘other’ category by physical therapists [62,63]. The higher use of manipulation by chiropractors (100%) is consistent with this intervention being at the core of chiropractic education. Explanations for the differences in the remaining categories were less dramatic and the reasons for them were less intuitive.

Similarities between the physical therapists and chiropractors were demonstrated in their utilization of electrotherapeutics, thermal agents, sonic agents, acupuncture, manual therapies, mechanical traction, ergonomic and work related interventions. Our review of the evidence has indicated that most of these categories have poor supporting evidence with the exception of manual therapies. The similarity in the use of manual therapies in general is not surprising since they are at the core of entry-level education for both professions. Reasons for their similarity in practice patterns with respect to the remaining interventions can only be speculated. It may be that a combination of factors is contributing to this practice pattern including caseload, patient expectations, infrastructure within the clinics, post-professional training, mentorship and others. For example, patients with neck pain may have a preference for heat applications and in busy private clinics supplementing the treatment sessions with heat may improve patient satisfaction and facilitate other aspects of the treatment program. Although workers compensation accounted for approximately one third of reimbursement in our respondents, it may be that practitioners are seeing patients who are having issues managing their neck pain at work even though the problem may not have directly resulted from a work injury. This along with practitioners implementing primary or secondary prevention strategies could explain the high use of ergonomic and work related interventions.

Overall the practice patterns demonstrated by this data suggest that chiropractors and physical therapists demonstrate strong utilization of interventions widely supported by the literature to manage neck pain. Interventions with limited or conflicting evidence have low to moderate use that is consistent with the uncertainty in the literature. The variability in use of interventions reflects the multimodal practice of practitioners that are commonly used to treat this population. Clinicians faced with applying the evidence-base must customize interventions to the presentation of the individual patient and we know that even within clinical trials response patterns vary across different patients. Therefore, some of the variability in practice patterns reflects this customization. There are multiple patient and practitioner preferences that affect the treatment choices made in clinician and patient interactions around managing neck pain. These can include previous experience, stage of healing, or practitioner type. However understanding the gaps between the evidence base and practice patterns is important for identifying areas needing targeted knowledge translation. Comparing current evidence with our survey results indicates that there are areas for education of chiropractors and physical therapists to increase utilization of interventions with supportive evidence. This includes low level laser and acupuncture therapy that were reported by only 13% and 45% respectively of our respondents. Our results also indicate an even greater need to educate practitioners about their use of interventions with weak supporting evidence such as work related and ergonomic interventions which were utilized by the majority of respondents. A greater understanding of explanatory factors of utilization may require further mixed methods research. As the evidence-based becomes clearer, it might be expected that practice patterns should become less variable.

To our knowledge our practice survey is the first to compare practice patterns of chiropractors and physical therapists for people with neck pain. Previous surveys pertaining to specific professions, reporting more broadly or on specific aspects of treatment have been published making comparison difficult. Within the physical therapy profession, surveys have focused on the utilization of manual therapies with greater attention to spinal (thrust) manipulation for neck pain. Internationally and in Canada, a decrease in manipulative procedures to the neck have been reported [64,65] and there is evidence of them generally being used less often in the neck compared to mobilization techniques [65-68]. Chiropractic surveys report much higher utilization of spinal (thrust) manipulative procedures [56,69,70], and this is not surprising since spinal manipulation is a core intervention within the paradigm of chiropractic practice. Chiropractors see a client base that consists mainly of people with back or neck pain [71] and are sought by the public for the treatment these problems [44,55]. In one population based survey, chiropractors and physical therapists were the second and third most utilized practitioners respectively for the treatment of chronic neck pain [55].

The effort to provide clear consistent messaging to relevant health professionals and the public regarding treatments with demonstrated effect as well as those without or even impeding recovery must continue and occur in multiple formats and mediums. This will help with the expenditure and appropriate allocation of healthcare dollars to minimize over-treatment with ineffective therapies or under-treatment of patients presenting with more complex conditions.

### Limitations

This survey has limitations that should be considered when interpreting the results. Our survey was cross-sectional and therefore provides a one-time perspective of general treatment trends. The benefit of this is that it can be revisited over time and results compared to detect change. We are also aware that our sample was not proportional and over-represented Canadian clinicians compared to other countries. It also was largely representative of the chiropractic and physical therapy professions. Our snowball sampling technique was limited by the associations known within our network and likely resulted in the exclusion of several relevant associations, that had we been able to sample, their input could have altered our results. Also we cannot be certain of our response rate due to the limitations of the information that we were able to receive from the organizations that distributed the survey link. We are aware of the potential for significant variation in the numbers of people that actually received the link and therefore the response rate provided is likely a very conservative one, yet still quite low. Those who choose to participate in survey research may represent a systematically different type of practitioner than those who don’t choose to participate. This survey was descriptive and to our knowledge the first of its kind. We did not conduct a Bonferroni correction to our results therefore allowing for the possibility that some reported differences in professions are due to chance. The results should not be considered generalizable but rather hypothesis generating to be further explored in future studies. Therefore, transferring the conclusions of this study to disciplines may not be appropriate.

## Conclusions

Our survey respondents indicated that they widely use interventions with a strong evidence base for effectiveness and that they also use a variety of other interventions with limited support or conflicting evidence. This suggests there is a need for research to fill gaps in evidence that are associated with variable practice patterns and knowledge translation to reduce the usage of some interventions that have been shown to be ineffective. Examining the consistency or lack of it between available evidence and current treatment patterns can influence guideline dissemination as well as other interventions, such as payment reform, to improve the effectiveness of current care for neck pain.

## Endnote

^a^LimeSurvey software, Survey Service & Consulting, Hamburg, Germany.

## Competing interests

The authors declare that they have no competing interests.

## Authors’ contributions

LC provided input at all stages of survey development, conducted the analysis, drafted the manuscript and incorporated feedback from all co-authors. JCM, ARG, DM and PLS provided input at all stages of survey development and provided feedback on the analysis and on the draft of the manuscript. All have given final approval for publication.

## ICON (International Collaboration on Neck)

This work was supported by Canadian Institutes of Health Research (CIHR) grant(s) FRN: KRS-102084 and FRN: 114380.

This work was supported by Canadian Institutes of Health Research (CIHR) grant(s) FRN: KRS-102084 and FRN: 114380.
